# Alpha-L-Fucosidase Has Diagnostic Value in Prostate Cancer With “Gray-Zone PSA” and Inhibits Cancer Progression *via* Regulating Glycosylation

**DOI:** 10.3389/fonc.2021.742354

**Published:** 2021-11-22

**Authors:** Cong Zhang, Jikai Liu, Fan Chao, Shiyu Wang, Dawei Li, Dunsheng Han, Zhonghua Xu, Guoxiong Xu, Gang Chen

**Affiliations:** ^1^ Department of Urology, Jinshan Hospital, Fudan University, Shanghai, China; ^2^ Department of Urology, Qilu Hospital, Shandong University, Jinan, China; ^3^ Research Center for Clinical Medicine, Jinshan Hospital, Fudan University, Shanghai, China

**Keywords:** prostate cancer, AFU, diagnosis, tumor progression, progression-free interval

## Abstract

**Background:**

This study aimed to explore the diagnostic value of alpha-l-fucosidase (AFU) in prostate cancer (PCa) patients with “gray-zone PSA” and to investigate the correlation between AFU expression and clinicopathological characteristics of PCa patients.

**Methods:**

The level of AFU and other necessary clinicopathological variables of patients were retrieved from electronic medical records. The transcriptome profiling and clinical information of PCa patients were obtained from The Cancer Genome Atlas (TCGA) database. The protein level of AFU in tissue was assessed by immunohistochemistry (IHC). All the data were processed by appropriate analysis methods. The p-value of <0.05 was considered statistically significant.

**Results:**

AFU showed ideal diagnostic value for PCa with prostate-specific antigen (PSA) levels ranging from 4 to 10 ng/ml, and its optimal cutoffs were 19.5 U/L. Beyond this, low AFU expression was associated with high pathological grade, T stage and N stage, more postoperative residual tumors, and poor primary therapy outcome, as well as shorter progression-free interval. The Kyoto Encyclopedia of Genes and Genomes (KEGG) analysis illustrated that FUCA1/FUCA2 exerted tumor-suppressive function by regulating the glycosylation.

**Conclusions:**

AFU (<19.5 U/L) could effectively distinguish the PCa from the patients with “gray-zone PSA”, and low expression of AFU was an independent unfavorable predictor for the clinicopathological characteristics of PCa patients.

## Introduction

Prostate cancer (PCa) is an epithelial malignancy with a high incidence that occurs in the male genitourinary system (1). In recent years, the morbidity and mortality of PCa have increased dramatically worldwide. Based on the last data, the morbidity of PCa ranks no. 1, and the mortality ranks no. 2 among male malignant tumors in 112 countries (2). In 2020, there were 1.4 million new PCa cases and 370,000 deaths globally (2). The treatment options of PCa vary based on cancer grade and stage. For example, surgery is the standard treatment for early PCa, and it can lead to a favorable prognosis. Therefore, early and accurate diagnosis is crucial for the treatment of PCa patients. Although the prostate-specific antigen (PSA) screening has made great contributions to the early diagnosis of PCa, some limitations are readily apparent. It is well established that the “diagnostic gray zone” existed in PSA screening due to the poor specificity (3). A prostate biopsy can provide an accurate diagnosis, but it is a time-consuming and expensive method that requires an experienced urologist and causes great suffering for patients. Therefore, the identification of effective and practical biomarkers for early and accurate diagnosis of PCa (especially men with a PSA of 4–10 ng/ml) is urgent and important.

AFU containing two isoforms, AFU1 and AFU2, is an enzyme that is capable of clearing the terminal α-l-fucose residues from glycoproteins (4, 5). AFU1 and AFU2 are encoded by *FUCA1* gene and *FUCA2* gene, respectively. Interestingly, high α-l-fucose expression has been reported to be correlated with many cancers, such as breast, thyroid, and colorectal cancers (6–10). Therefore, it is reasonable to speculate that AFU with the function of hydrolyzing α-l-fucose may imply tumor-suppressive function. Previous studies have confirmed that AFU is indeed lowly expressed in a variety of cancers, including colon cancer, colorectal cancer, and breast cancer (11–13). Other than that, low expression of AFU usually predicts a worse prognosis in cancer patients (12–14).

However, the possible correlations between AFU and PCa have not yet been explored. Consequently, the current study is conducted to investigate the relationship between AFU expression and PCa. We hope that the current research can identify a promising early diagnostic and effective prognostic biomarker for PCa.

## Materials and Methods

### Patients

Patients’ clinicopathological information was collected and analyzed through retrospective chart reviews of electronic medical records of Qilu Hospital of Shandong University between 2013 and 2020. Following inclusion and exclusion criteria, a total of 106 PCa patients with PSA levels between 4 and 10 ng/ml met the requirements. Those patients all accepted prostate biopsy and were confirmed as PCa by biopsy pathological results. Meanwhile, 113 benign prostatic hyperplasia (BPH) patients whose PSA levels ranged from 4 to 10 ng/ml were included as a control in this retrospective study. Beyond that, in order to further investigate the relationship between AFU expression and clinicopathological features of PCa patients, 196 eligible PCa patients who were treated with radical prostatectomy at Qilu Hospital were integrated into the current study.

The inclusion criteria were the following:
1) Necessary information was available, such as important test records, clinicopathological variables, and other necessary data.
2) The postoperation pathological outcomes indicated benign prostatic hyperplasia or prostatic adenocarcinoma.
The exclusion criteria were the following:
1) Coexisting other malignant diseases or history of tumor or cancer
2) Suffering from immune system disease or hematologic disorders
3) Taking procoagulant or anticoagulant or other medicine interfering with lab test within the past 2 weeks

### Data Collection

Essential demographic information, important laboratory results, and clinicopathological data were retrieved from electronic patient records. The pathological grade was evaluated using the Gleason system. It was divided into two groups: high-pathological grade group (Gleason scores ≥8) and low-pathological grade group (Gleason scores <8) as described by a previous study (15). The stage was judged by the 2002 TNM classification (16).

### Alpha-l-Fucosidase Measurement

After 12-h fasting, 5 ml of venous blood was drawn from each patient before he received any clinical treatment in the early morning. Blood was stored in the blood-sampling tubes containing procoagulant. Subsequently, samples were centrifuged at 2,000 rpm for 10 min, and the serum was separated to determine the activity of AFU by The Roche Cobas 8000 automatic analyzer (Roche, Switzerland) according to the standard operating procedure.

### Extraction and Analysis of The Cancer Genome Atlas Datasets

The transcriptome profiling and clinical information of PCa patients were obtained from The Cancer Genome Atlas (TCGA) database (https://www.cancer.gov/tcga). The expression of *FUCA1*/*FUCA2* and important clinicopathological variables of PCa patients, such as pathological grade, stage, and survival data, were extracted and analyzed.

### Immunohistochemistry Analysis Based on The Human Protein Atlas

The protein expression of AFU in PCa tissue was evaluated under the support of the online website The Human Protein Atlas (https://www.proteinatlas.org/).

### Statistical Analysis

The expression of AFU in each group was shown as mean and SD. The correlations between AFU levels and variables were assessed by Student’s t-test if the data followed a normal distribution, and if not, using the Mann–Whitney test. The data were obtained from TCGA by employing chi-square test, Fisher’s exact test, or Wilcoxon rank-sum test. Kaplan–Meier and Cox regression methods were used to evaluate the survival data from TCGA. The hazard ratio (HR) and 95% CI were calculated by logistic regression or Cox regression model. A two-sided *p*-value was set in the current study, and a *p*-value of <0.05 was regarded as statistically significant. Statistical analysis was performed by the Statistical Package for Social Sciences version 20.0 (SPSS Inc., Chicago, IL, USA), GraphPad Prism 8 software (GraphPad Software Inc., San Diego, CA, USA), and R (version 3.6.3; R Foundation, Vienna, Austria).

## Results

### Baseline Clinicopathological Characteristics

**Figure 1:**
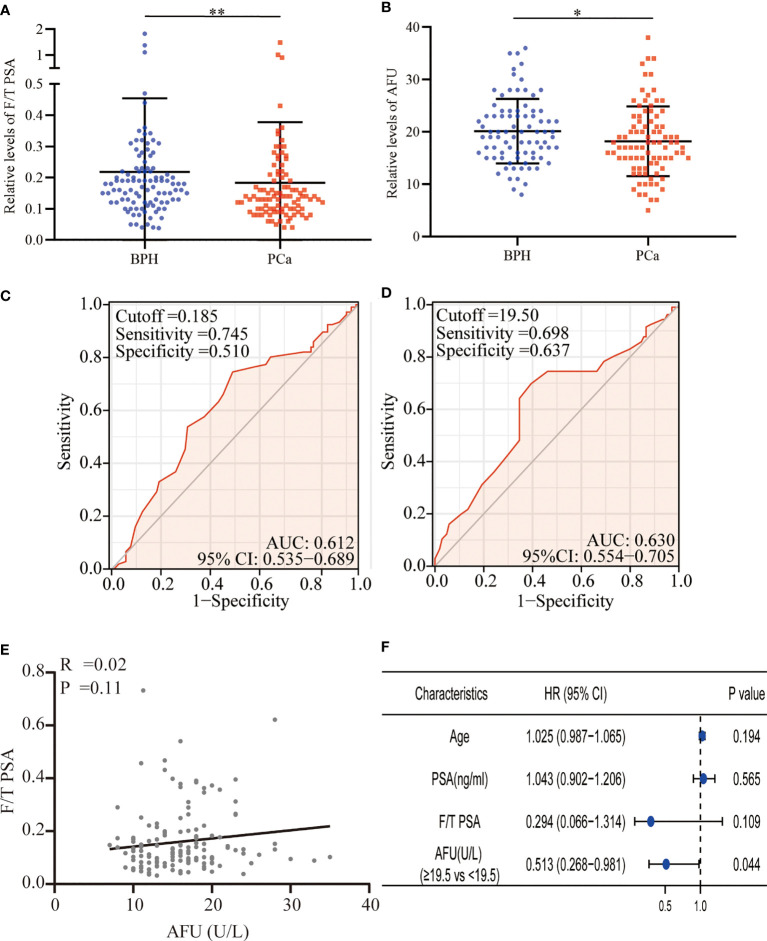
F/T PSA and AFU level in BPH and PCa. **(A)** F/T PSA level. **(B)** AFU level. **(C)** The diagnostic value of F/T PSA for PCa. **(D)** The diagnostic value of AFU for PCa. **(E)** The line correlation between F/T PSA and AFU. **(F)** Logistic analysis of AFU expression for the PCa diagnosis; *p < 0.05, **p < 0.01. F/T PSA, free/total prostate-specific antigen; AFU, alpha-l-fucosidase; BPH, benign prostatic hyperplasia; PCa, prostate cancer.

A total of 219 patients with PSA between 4 and 10 ng/ml were enrolled in the present study. Based on the pathological results after a needle biopsy, 113 patients were diagnosed with BPH, and 106 patients were confirmed to have PCa. Surprisingly, the mean level of PSA in the BPH was higher than that in the PCa, although no statistical differences were presented (7.84 ± 2.55 vs. 7.48 ± 2.58 ng/ml, *p* = 0.675). It was meaningful that we found that the free/total (F/T) PSA and AFU levels in the BPH patients were higher than in the PCa patients (F/T PSA: 0.22 ± 0.24 vs. 0.18 ± 0.19, *p* = 0.008; AFU: 20.16 ± 6.17 U/L vs. 18.21 ± 6.66 U/L, *p* = 0.049) (**Table 1** and **Figures 1A, B**), but there was no line correlation between them (**Figure 1E**). Then, the receiver operating characteristic (ROC) analysis indicated that AFU had a better value for PCa diagnosis than F/T PSA especially in specificity (the area under the ROC curve (AUC): 0.630 vs. 0.612) (**Figures 1C, D**), and the AFU optimal cutoffs for PCa was 19.5 U/L. Logistic regression analysis was employed to further validate the AFU cutoffs’ diagnostic value for PCa, and the results indicated that AFU cutoffs showed ideal diagnostic performance for PCa (≥19.5 vs. <19.5 U/L: HR = 0.513, *p* = 0.044) (**Figure 1F**).

**Table 1:** Clinical characteristics of PCa and BPH patients.

Characteristics	BPH	PCa	*p-*Value
Patients (n; %)	113 (51.6%)	106 (48.4%)	
Age (years)	68.08 ± 8.99	69.97 ± 8.43	0.149^*^
PSA (ng/ml)	7.84 ± 2.55	7.48 ± 2.58	0.675^*^
F/T PSA	0.22 ± 0.24	0.18 ± 0.19	0.008^*^
AFU (U/L)	20.16 ± 6.17	18.21 ± 6.66	0.049^*^

p < 0.05 is considered as statistically significant.

PCa, prostate cancer; BPH, benign prostatic hyperplasia; PSA, prostate-specific antigen; F/T PSA, free/total prostate-specific antigen; AFU, alpha-l-fucosidase.

*p: Mann–Whitney U-test.

### Associations Between Alpha-l-Fucosidase Expression and Clinicopathological Variables of 196 Prostate Cancer Patients

**Figure 2:**
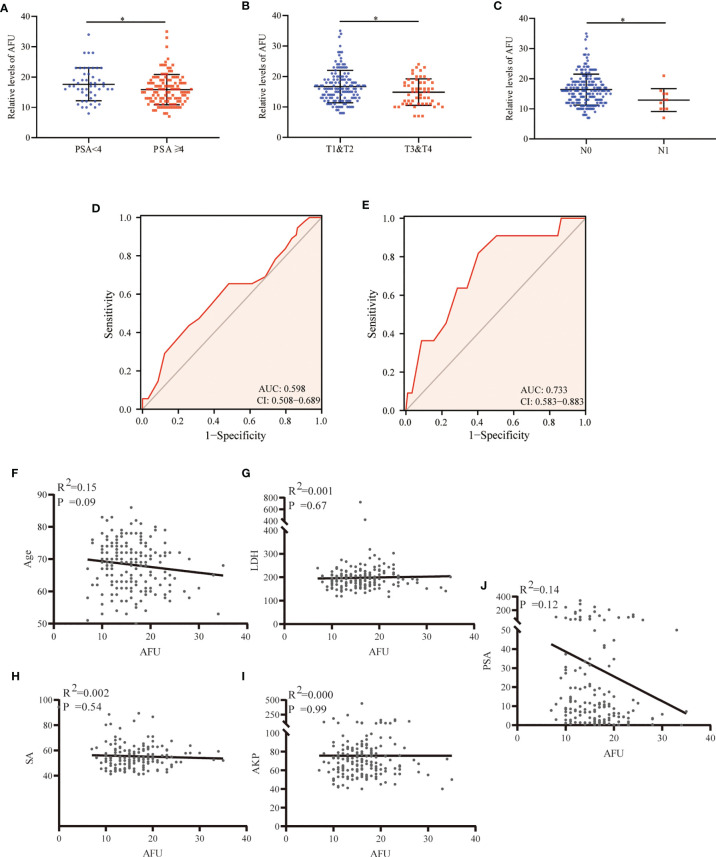
AFU expression in PCa subgroups. **(A)** PSA. **(B)** T stage. **(C)** N stage. **(D)** The diagnostic value of AFU for advanced T stage. **(E)** The diagnostic value of AFU for lymph node metastasis. Line correlations among AFU and PCa patient’s variables: **(F)** age; **(G)** LDH; **(H)** SA; **(I)** AKP; and **(J)** PSA. *p < 0.05. AFU, alpha-l-fucosidase; PCa, prostate cancer; PSA, prostate-specific antigen; LDH, lactate dehydrogenase; SA, serum sialic acid; AKP, alkaline phosphatase.

A total of 196 PCa patients who underwent radical prostatectomy, with the PSA average of 30.30 ng/ml (ranking 0.04 to 343 ng/ml), were analyzed in the present study. The results indicated that AFU expression was inversely correlated with PSA. The PCa patients with higher PSA levels (PSA ≥ 4 ng/ml) indicated lower AFU expression (15.89 ± 5.01 U/L) and vice versa (*p* = 0.043) (**Table 2** and **Figure 2A**). Importantly, non-localized PCa (pT3 and pT4) showed lower AFU expression than localized PCa (pT1 and pT2) (*p* = 0.05) (**Table 2** and **Figure 2B**). Likewise, the advanced PCa with lymph node metastasis had a lower AFU level than that without lymphatic metastasis (*p* = 0.017) (**Table 2** and **Figure 2C**). Although differences were not statistically significant, we found that the high pathological grade group had lower AFU levels than the low pathological grade group (**Table 2**). The results of the ROC analyses indicated good predictive power of AFU for PCa pathological T stage and N stage especially for N stage (**Table 2** and **Figures 2D, E**). However, no linear relationship was observed among AFU levels and age, lactate dehydrogenase (LDH), serum sialic acid (SA), alkaline phosphatase (AKP), and PSA (**Figures 2F–J**).

**Table 2:** Correlations between preoperative AFU levels and clinicopathological parameters of PCa patients.

Characteristics	N (%)	AFU levels (U/L, mean ± SD)	*p-*Value
**Patients^a^**	196 (100%)	16.35 ± 5.20	
**Age**			0.144*
<69	92 (46.9%)	16.92 ± 5.87	
≥69	104 (53.1%)	15.59 ± 4.29	
**PSA (ng/**ml**)**			**0.043** *
<4	49 (25%)	17.63 ± 5.41	
≥4	116 (59.2%)	15.89 ± 5.01	
Missing data	31 (15.8%)	–	
**LDH^a^**			0.585*
<197	102 (52.0%)	16.47 ± 5.42	
≥197	80 (40.8%)	16.41 ± 4.60	
Missing data	14 (7.1%)	–	
**SA^a^**			0.954*
<56	110 (56.1%)	16.38 ± 4.83	
≥56	71 (36.2%)	16.62 ± 5.44	
Missing data	15 (7.7%)	–	
**AKP^a^**			0.364*
<75	114 (58.2%)	16.34 ± 5.58	
≥75	71 (36.2%)	16.42 ± 4.11	
Missing data	11 (5.6%)	–	
**Pathological grade group**			
Low-grade group (<8)	107 (54.6%)	16.45 ± 5.31	0.531*
High-grade group (≥8)	89 (45.4%)	15.93 ± 4.90	
**T stage**			**0.050** *
T1 and T2	141 (71.9%)	16.73 ± 5.32	
T3 and T4	55 (28.1%)	14.89 ± 4.35	
**N stage**			**0.017** *
N0	185 (94.4%)	16.41 ± 5.13	
N1	11 (5.6%)	12.91 ± 3.81	
**Bone metastasis**			0.105^a^ *
No	99 (50.5%)	16.74 ± 5.36	0.924^b^ *
Yes	14 (7.1%)	14.21 ± 4.14	
Suspicion	23 (11.7%)	16.30 ± 4.70	
Missing data	60 (30.6%)	–	

Pathological grade falls into high grade and low grade using the GS. Pathological stage is assessed by postoperative pathology results (not biopsy) in accordance with 2002 TNM classification; p < 0.05 is considered as statistically significant.

PCa, prostate cancer; BPH, benign prostate hyperplasia; AFU, alpha-l-fucosidase; PSA, prostate-specific antigen; LDH, lactate dehydrogenase; SA, serum sialic acid; AKP, alkaline phosphatase; GS, Gleason system.

^a^Continuous variables are expressed as median.

^a^p: no bone metastases versus bone metastases.

^b^p: no bone metastases versus suspicion.

*p: Mann–Whitney U-test.

Bold values was used for emphasis, means p ≤ 0.05.

### *FUCA1* and *FUCA2* Expression in Prostate Cancer Based on The Cancer Genome Atlas Database

**Figure 3:**
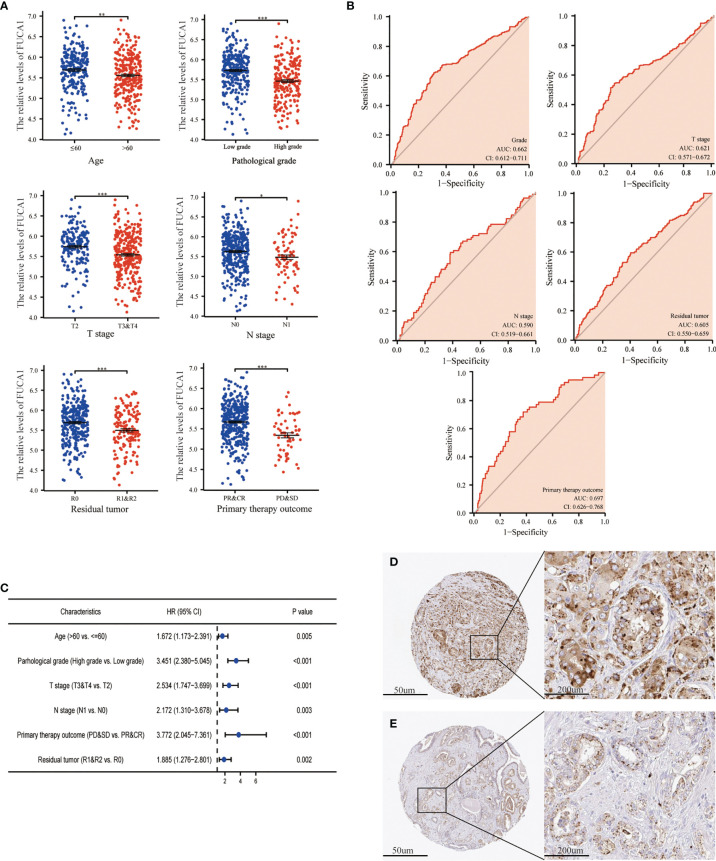
The expression of *FUCA1* in PCa subgroups. **(A)** *FUCA1* mRNA expression in different PCa subgroups. **(B)** The diagnostic value of *FUCA1* for different PCa clinicopathological parameters. **(C)** Logical regression analysis of clinicopathological variables’ effects on low *FUCA1* expression. IHC analysis of *FUCA1* in low- **(D)** and high-grade **(E)** PCa tissue. CR, complete response; PR, partial response; SD, stable disease; PD, progressive disease. *p < 0.05, **p < 0.01, ***p < 0.01. PCa, prostate cancer; IHC, immunohistochemistry.

**Figure 4:**
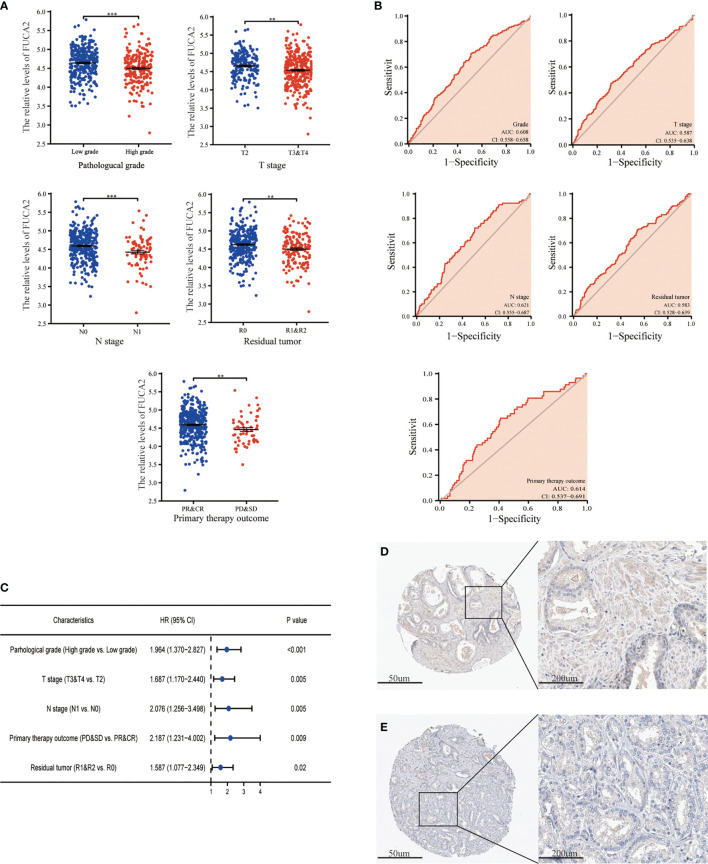
The expression of *FUCA2* in PCa subgroups. **(A)** *FUCA2* mRNA expression in different PCa subgroups. **(B)** The diagnostic value of *FUCA2* for different PCa clinicopathological parameters. **(C)** Logical regression analysis of clinicopathological variables’ effects on low *FUCA2* expression. IHC analysis of *FUCA2* in low- **(D)** and high-grade **(E)** PCa tissue. CR, complete response; PR, partial response; SD, stable disease; PD, progressive disease. **p < 0.01, ***p < 0.01. PCa, prostate cancer; IHC, immunohistochemistry.

The expression of *FUCA1*/*FUCA2* and clinicopathological data of 499 PCa patients were extracted from TCGA database and presented in **Tables 3**, **4**, respectively. *FUCA1* expression was lower in the older group (*p* = 0.0006) compared with the younger group (**Table 3** and **Figure 3A**), but *FUCA2* expression had no notable difference between the two groups (*p* = 0.896) (**Table 4**). Notably, lower *FUCA1*/*FUCA2* expression predicted both higher pathological grade group and stage, more residual tumors, and worse therapeutic effect (**Figures 3A**, **4A**). In parallel, the ROC analysis confirmed that low *FUCA1* and *FUCA2* indeed promoted PCa progression, metastasis, and drug resistance (**Figures 3B**, **4B**). All the above results were further validated by logistic regression analysis (**Figures 3C**, **4C**).

**Table 3:** Correlations between FUCA1 expression and clinicopathological parameters of PCa patients.

Characteristic	Low expression of FUCA1 n (%)	High expression of FUCA1 n (%)	*p-*Value
**Patients**	249 (40.9%)	250 (50.1%)	
**Age**			0.006^***^
≤60	96 (19.2%)	128 (25.7%)	
>60	153 (30.7%)	122 (24.4%)	
**PSA (ng/ml)**			0.092^*^
<4	199 (45%)	216 (48.9%)	
≥4	18 (4.1%)	9 (2%)	
**Pathological grade group**			<0.001^*^
Low-grade group (Gleason score < 8)	110 (22%)	183 (36.7%)	
High-grade group (Gleason score ≥ 8)	139 (31%)	67 (13.4%)	
**T stage**			<0.001^*^
T2	68 (13.8%)	121 (24.6%)	
T3 and T4	178 (36.2%)	125 (25.4%)	
**N stage**			0.004^*^
N0	168 (39.4%)	179 (42%)	
N1	53 (12.4%)	26 (6.1%)	
**M stage**			0.621^**^
M0	225 (49.1%)	230 (50.2%)	
M1	2 (0.4%)	1 (0.2%)	
**Primary therapy outcome**			<0.001^*^
PR and CR	171 (39.1%)	210 (48%)	
PD and SD	43 (9.9%)	14 (3.2%)	
**Residual tumor**			<0.001^**^
R0	140 (29.9%)	175 (37.4%)	
R1 and R2	92 (19.6%)	61 (13.1%)	
**OS event, n (%)**			0.063^**^
Alive	241 (48.3%)	248 (49.7%)	
Dead	8 (1.6%)	2 (0.4%)	
**DSS event, n (%)**			0.684^**^
Alive	244 (49.1%)	248 (49.9%)	
Dead	3 (0.6%)	2 (0.4%)	
**PFI event, n (%)**			<0.001^*^
Alive	184 (36.9%)	221 (44.3%)	
Dead	65 (13%)	29 (5.8%)	

p < 0.05 is considered as statistically significant.

PCa, prostate cancer; BPH, benign prostate hyperplasia; OS, overall survival; DSS, disease-specific survival; PFI, progression-free interval; PR, partial response; CR, complete response; PD, progressive disease; SD, stable disease.

^*^p: chi-square test.

^**^p: Fisher’s test.

^***^p: Wilcoxon rank-sum test.

**Table 4:** Correlations between FUCA2 expression and clinicopathological parameters of PCa patients.

Characteristic	Low expression of FUCA2 n (%)	High expression of FUCA2 n (%)	*p*-Value
**Patients**	249 (40.9%)	250 (50.1%)	
Age			0.896^***^
≤60	113 (22.6%)	111 (22.2%)	
>60	136 (27.3%)	139 (27.9%)	
**PSA (ng/ml)**			0.112^*^
<4	203 (45.9%)	212 (48%)	
≥4	18 (4.1%)	9 (2%)	
**Pathological grade group**			0.001^*^
Low-grade group (Gleason score < 8)	126 (25.28%)	167 (33.5%)	
High-grade group (Gleason score ≥ 8)	123 (24.6%)	83 (16.6%)	
**T stage**			0.005^*^
T2	79 (16.1%)	110 (22.4%)	
T3 and T4	166 (33.7%)	137 (27.8%)	
**N stage**			0.007^*^
N0	167 (39.2%)	180 (42.3%)	
N1	52 (12.2%)	27 (6.3%)	
**M stage**			0.618^**^
M0	232 (50.7%)	223 (48.7%)	
M1	1 (0.2%)	2 (0.4%)	
**Primary therapy outcome**			0.008^*^
PR and CR	182 (41.6%)	199 (45.5%)	
PD and SD	38 (8.6%)	19 (4.4%)	
**Residual tumor**			0.023^*^
R0	145 (31%)	170 (36.3%)	
R1 and R2	88 (18.8%)	65 (13.9%)	
**OS event**			0.751^**^
Alive	245 (49.1%)	244 (48.9%)	
Dead	4 (0.8%)	6 (1.2%)	
**DSS event**			0.373^**^
Alive	246 (49.5%)	246 (49.5%)	
Dead	1 (0.2%)	4 (0.8%)	
**PFI event**			0.049^*^
Alive	193 (38.7%)	212 (42.5%)	
Dead	56 (11.2%)	38 (7.6%)	

p < 0.05 is considered as statistically significant.

PCa, prostate cancer; BPH, benign prostate hyperplasia; OS, overall survival; DSS, disease-specific survival; PFI, progression-free interval; PSA, prostate-specific antigen; PR, partial response; CR, complete response; PD, progressive disease; SD, stable disease.

^*^p: chi-square test.

^**^p: Fisher’s test.

^***^p: Wilcoxon rank-sum test.

### Immunohistochemistry Staining of Alpha-l-Fucosidase

AFU protein levels in PCa tissue were further measured by immunohistochemistry (IHC) staining based on the online website, The Human Protein Atlas (https://www.proteinatlas.org/). As may be immediately apparent, the expression of AFU1 and AFU2 were much lower in high-grade PCa tissue compared with low-grade tissue (**Figures 3D, E** and **4D, E**).

### Low Expression of *FUCA1*/*FUCA2* Predicted Worse Prognosis of Prostate Cancer Patients

**Figure 5:**
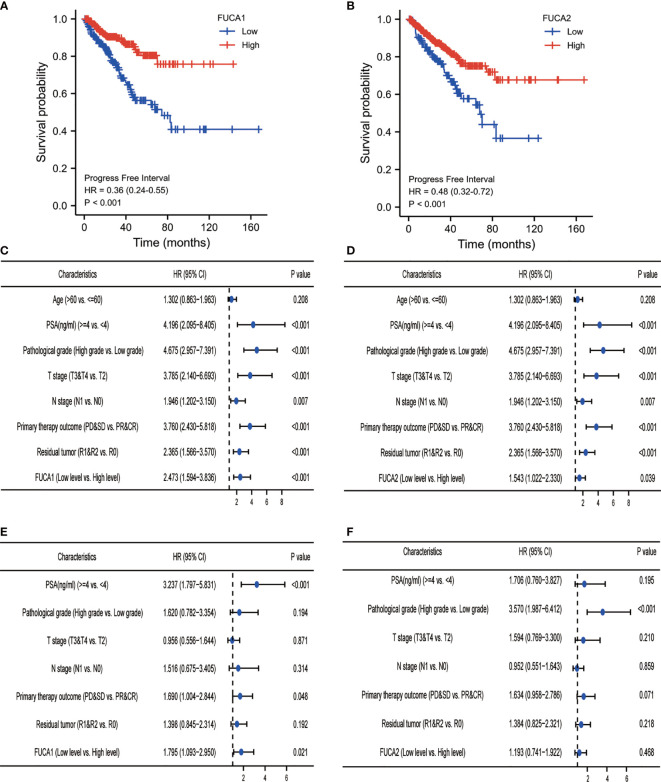
Survival analysis of PCa patients with different *FUCA1*/*FUCA2* expression based on TCGA databases. The Kaplan–Meier curve analysis based on *FUCA1* **(A)** and *FUCA2* **(B)** expression effect for PFI. Univariate Cox regression analysis of *FUCA1* **(C)** and *FUCA2* **(D)** expression effect for PFI. Multivariate Cox regression analysis of *FUCA1* **(E)** and *FUCA2* **(F)** expression effect for PFI. PCa, prostate cancer; TCGA, The Cancer Genome Atlas; PFI, progress-free interval.

Log-rank analysis indicated that the lower level of *FUCA1*/*FUCA2* indicated shorter progression-free interval (PFI) of PCa patients (**Figures 5A, B**). Cox regression model illustrated that low *FUCA1* expression was a reliable indicator for PCa patients’ poor prognosis (**Figures 5C, E**), but the prognostic performance of *FUCA2* was susceptible to other factors (**Figures 5D, F**).

### Kyoto Encyclopedia of Genes and Genomes Analysis Indicated *FUCA1*/*FUCA2* Exerted Biological Function Through Regulating Glycosylation

**Figure 6:**
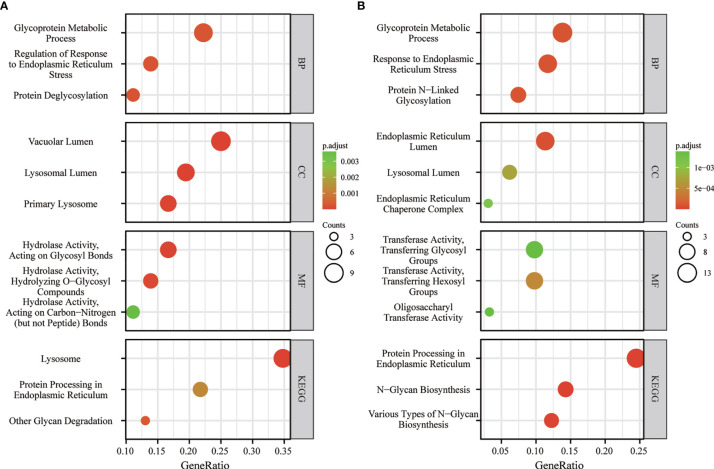
The KEGG analysis of *FUCA1* **(A)** and *FUCA2* **(B)**. KEGG, The Kyoto Encyclopedia of Genes and Genomes.

In order to probe the underlying mechanism through which *FUCA1*/*FUCA2* exerted its functional role, the Kyoto Encyclopedia of Genes and Genomes (KEGG) analysis was applied. The analysis results indicated that *FUCA1* and *FUCA2* both played an essential role in the regulation of glycosylation, especially in the protein glycosylation (**Figures 6A, B**).

## Discussion

The present study indicates, for the first time, that AFU can effectively distinguish PCa from patients with PSA levels ranging from 4 to 10 ng/ml. We find that compared with the BPH patients, the PCa patients have lower serum AFU expression and smaller values of F/T PSA, both with gray-zone PSA level. We are aware that PSA is secreted by prostate epithelial cells, and its level will be elevated in PCa and BPH (17). Therefore, it is difficult to distinguish early PCa from BPH solely dependent on the PSA expression. In line with a previous study (18), our results indicated that no meaningful difference in PSA levels was observed between BPH and PCa patients with “gray-zone PSA”. To validate and further test the diagnostic reliability of F/T PSA and AFU, the logistic regression analysis was applied. However, the results illustrated that only AFU but not F/T PSA still exhibited a robust and independent diagnostic value for PCa. Likewise, the ROC analysis indicated that the diagnostic efficiency of F/T PSA was inferior compared with that of AFU. These data indicated that the diagnostic value of F/T PSA was more vulnerable to be interfered with other factors such as age and PSA level; for this reason, F/T PSA was not a reliable indicator for PCa patients with gray-zone PSA level. After confirmation of the diagnostic value of AFU for PCa, the possible correlations between AFU expression and PCa patients’ clinicopathological varies, which were further explored. Consistent with the above conclusion, lower expression of AFU implied a worse outcome. Compared with those of the early-stage group (pT1 and pT2 stages), the levels of AFU were markedly decreased in the advanced stage group (pT3 and pT4 stages). Furthermore, the patients with lymph node metastases had lower expression of AFU than those without lymph node metastases. The expression of AFU in prostate tissue was assessed using IHC. Similarly, it indicated the AFU expression was lower in high-grade PCa in contrast to low-grade PCa.

All the above analyses were conducted for AFU protein; next, the mRNA level of AFU was further evaluated based on TCGA database. Previous studies report AFU containing two subtypes, AFU1 and AFU2, which are encoded by genes *FUCA1* and *FUCA2*, respectively (5, 19, 20). Therefore, the relationships between *FUCA1*/*FUCA2* expression and PCa patients’ clinicopathological characteristics were further assessed based on TCGA database. It has long been known that the incidence of malignant disease in human is rapidly increased with aging, while our result suggested that the *FUCA1* level was decreased with the increase of age. Beyond that, we found significant correlations among *FUCA1*/*FUCA2* expression and pathological grade, pathological stage, postoperative residual tumor numbers, and primary therapeutic effect. Although *FUCA1* and *FUCA2* both showed prognostic value for patients’ PFI, the diagnostic performance of *FUCA1* is more accurate and stable than that of *FUCA2*. All of these results imply that *FUCA1*/*FUCA2* may be acting in a tumor-suppressive role, and lower expression of *FUCA1*/*FUCA2* prognosticates worse pathological results, less therapeutic effect, and shorter PFI. This finding is consistent with many previous studies, which further validate the reliability of our conclusion (12, 13, 21–23).

The KEGG analysis indicated the biological function of *FUCA1* and *FUCA2* mainly involved glycosylation, especially glycoprotein. Glycosylation plays an important role in the initiation and progression of human disease including infection, inflammation, metabolism, and, of course, tumors (24–26). Some well-known tumor markers such as haptoglobin and CA 19-9 are fucosylated glycoproteins (27, 28). Apart from this, several key signal proteins, like integrin, E-cadherin, TGF-β receptors, and epidermal growth factor receptor (EGFR), are glycoproteins as well, which indicates that modification of glycosylation has a complex and crucial effect on their functions (29–32). Remarkably, many studies have revealed that abnormal glycosylation can lead to tumor onset and progression (33–36). AFU encoded by *FUCA1* or *FUCA2* can remove the terminal fucose residues from glycans and prevents aberrant accumulation of fucose-containing glycans (5, 26). Thus, the lack of AFU, which is responsible for the degradation of glycans, causes the overexpression of glycans and may prompt tumor initiation and development.

In this study, we first showed that AFU could be an effective diagnostic marker for PCa patients who had “gray-zone PSA”. In addition, our study demonstrated that low expression of AFU portends a worse prognosis of PCa. However, some limitations that existed in the present study deserve special attention. First, our study is retrospective research, which only allows for speculation based on the available data. Second, in consideration of the longer survival time of PCa patients, the differences in survival among different subgroups are difficult to be analyzed. Third, by bioinformatics analysis, we speculated that AFU suppressed the progression of PCa *via* regulation of glycosylation metabolism, which awaited further experimental validation.

## Conclusion

AFU can effectively distinguish PCa from patients with gray-zone PSA levels; and lower AFU expression predicates advanced pathological results, poor therapeutic effect, more postoperative residual tumor numbers, and worse prognosis of PCa patients.

## Data Availability Statement

The original contributions presented in the study are included in the article/**Supplementary Material**. Further inquiries can be directed to the corresponding authors.

## Ethics Statement

The studies involving human participants were reviewed and approved by the Research Ethics Committee of Qilu Hospital of Shandong University. The patients/participants provided their written informed consent to participate in this study.

## Author Contributions

Study design: GC and ZX. Data collection: CZ, JL, DL, and ZX. Writing: CZ, GX, and GC. Editing: CZ, FC, SW, and DH. All authors contributed to the article and approved the submitted version.

## Funding

This work was supported by grants from the National Science Foundation of Shanghai (No. 18ZR1405800) and the Project for Key Medical Specialty Construction in Jinshan District (6th Period, Type A) (No. JSZK2019A03) to GC.

## Conflict of Interest

The authors declare that the research was conducted in the absence of any commercial or financial relationships that could be construed as a potential conflict of interest.

## Publisher’s Note

All claims expressed in this article are solely those of the authors and do not necessarily represent those of their affiliated organizations, or those of the publisher, the editors and the reviewers. Any product that may be evaluated in this article, or claim that may be made by its manufacturer, is not guaranteed or endorsed by the publisher.

## Abbreviations

PCa, prostate cancer; BPH, benign prostatic hyperplasia; AFU, alpha-l-fucosidase; PSA, prostate-specific antigen; SA, serum sialic acid; LDH, lactate dehydrogenase; AKP, alkaline phosphatase; HR, hazard ratios; PFI, progression-free interval.
